# Early Stabilization Does Not Increase Complication Rates in Acetabular Fractures of the Elderly: A Retrospective Analysis from the German Pelvis Registry

**DOI:** 10.3390/jcm12227043

**Published:** 2023-11-11

**Authors:** Stephan Regenbogen, Iris Leister, Alexander Trulson, Lisa Wenzel, Jan Friederichs, Fabian M. Stuby, Andreas Höch, Markus Beck, Working Group on Pelvic Fractures of the German Trauma Society

**Affiliations:** 1 Department of Traumatology and General Surgery, Berufsgenossenschaftliche Unfallklinik Murnau, 82418 Murnau, Germany; s_regenbogen@web.de (S.R.); alexander.trulson@bgu-murnau.de (A.T.); lisa.wenzel@bgu-murnau.de (L.W.); jan.friederichs@bgu-murnau.de (J.F.); fabian.stuby@bgu-murnau.de (F.M.S.); 2 Department of Traumatology, Berufsgenossenschaftliche Unfallklinik Ludwigshafen, 67071 Ludwigshafen, Germany; 3 Spinal Cord Injury Center, Berufsgenossenschaftliche Unfallklinik Murnau, 82418 Murnau, Germany; iris.leister@bgu-murnau.de; 4 Department for Orthopaedics, Trauma Surgery and Plastic Surgery, University Hospital of Leipzig, 04103 Leipzig, Germany; andreas.hoech@medizin.uni-leipzig.de

**Keywords:** acetabular fractures, complications, geriatric patients, registry study, timing of surgery

## Abstract

***Background***: The incidence of acetabular fractures in geriatric patients has increased. Although there are strong data supporting the early operative treatment of hip fractures in geriatric patients, the optimal timing for acetabular fractures remains unclear and for several reasons, delayed treatment after trauma is common. ***Methods***: A retrospective analysis of the German Pelvis Registry between 2008 and 2017 was performed. Ultimately, 665 patients with fractures of the anterior column or anterior column and posterior hemitransverse were enrolled. Patients above and below 65 years of age with these fracture types were analyzed regarding surgery day (within 48 hours, between 2 and 4 days, after 4 days), complication rate, reduction quality, and hospital stay. ***Results***: The complication rate of the geriatric group was twice as high as that of younger patients; however, this finding was independent of the timing of surgery. Reduction quality and hospital stay were independent of surgical timing. ***Conclusions***: In contrast to other fracture types, such as proximal femur fractures, the timing of surgery for acetabular fractures does not have a significant impact on the patient’s outcome. The optimal time for surgery cannot be determined using the current data. However, as expected, there is a significantly higher risk for postoperative complications in the geriatric population.

## 1. Introduction

Proximal femur fractures, also known as hip fractures, are a common type of injury, especially among the elderly population. Because of their high incidence, there has been a significant amount of research conducted on the optimal timing of surgery for these types of fractures. These studies have consistently shown that early surgical intervention can lead to improved outcomes, faster recovery times, and a reduced risk of complications [1]. Therefore, for proximal femur fractures, there are clear recommendations for prompt surgical treatment within 24 hours [2,3,4,5,6,7].

Today’s surgeons are confronted with a constantly increasing number of acetabular fractures, especially in geriatric patients [8,9,10,11]. The vast majority of patients suffering low-energy acetabular fractures are part of the same patient population that is at risk for proximal femur fractures. Acetabular fractures can result in significant disability and decreased quality of life if not treated appropriately [1,12]. The restoration of joint congruency is of crucial importance [13,14]. Despite the rapidly rising incidence of acetabular fractures, especially in the elderly population, there is a lack of data focusing on the optimal timing of surgery. Although there is some evidence to suggest that early surgical intervention can lead to improved outcomes [15,16,17,18,19,20], the optimal timing remains unclear, and management is mainly based on clinical experience.

Acetabular fractures in geriatric patients mostly occur because of minor trauma like ground-level falls. Due to reduced bone quality, the two most represented fracture types in elderly patients are the fracture of the anterior column and the anterior column with associated posterior hemitransverse fracture [9,10]. These fractures rarely lead to significant hip joint dislocations, and therefore, immediate surgery is seldom required. Because the approach to these fractures is an anterior intrapelvic approach, there is an increased risk of bleeding. To reduce the risk of bleeding, operative treatment is often considered with a delay of 2–4 days. This delay is accepted under the idea that blood clotting can achieve hemostasis, resulting in reduced intraoperative blood loss and better visibility during the procedure [21,22]. Furthermore, because of its rarity and the complexity of the procedure, surgical experience is needed, and treatment should only be performed in specialized departments, a fact that might temporarily delay surgical treatment [23,24].

The aim of this study was to retrospectively evaluate the data from the German Pelvis Registry to determine if an optimal time for surgery of acetabular fractures could be detected.

## 2. Materials and Methods

In this cohort study, the data from the German Pelvis Registry (GPR) were analyzed retrospectively. The GPR is a prospective nationwide multicenter database with up to 32 participating hospitals in Germany. Eligibility criteria for enrollment into the registry are a pelvic ring and/or acetabular fracture and informed consent of the patient. The Ethics Committee of the Chamber of Physicians of the Federal State of Saarland approved the GPR (No. 29/14). As a retrospective analysis of anonymized data, there was no need for further approval from the local ethics committee.

### 2.1. Data Source and Study Design

**Figure 1:**
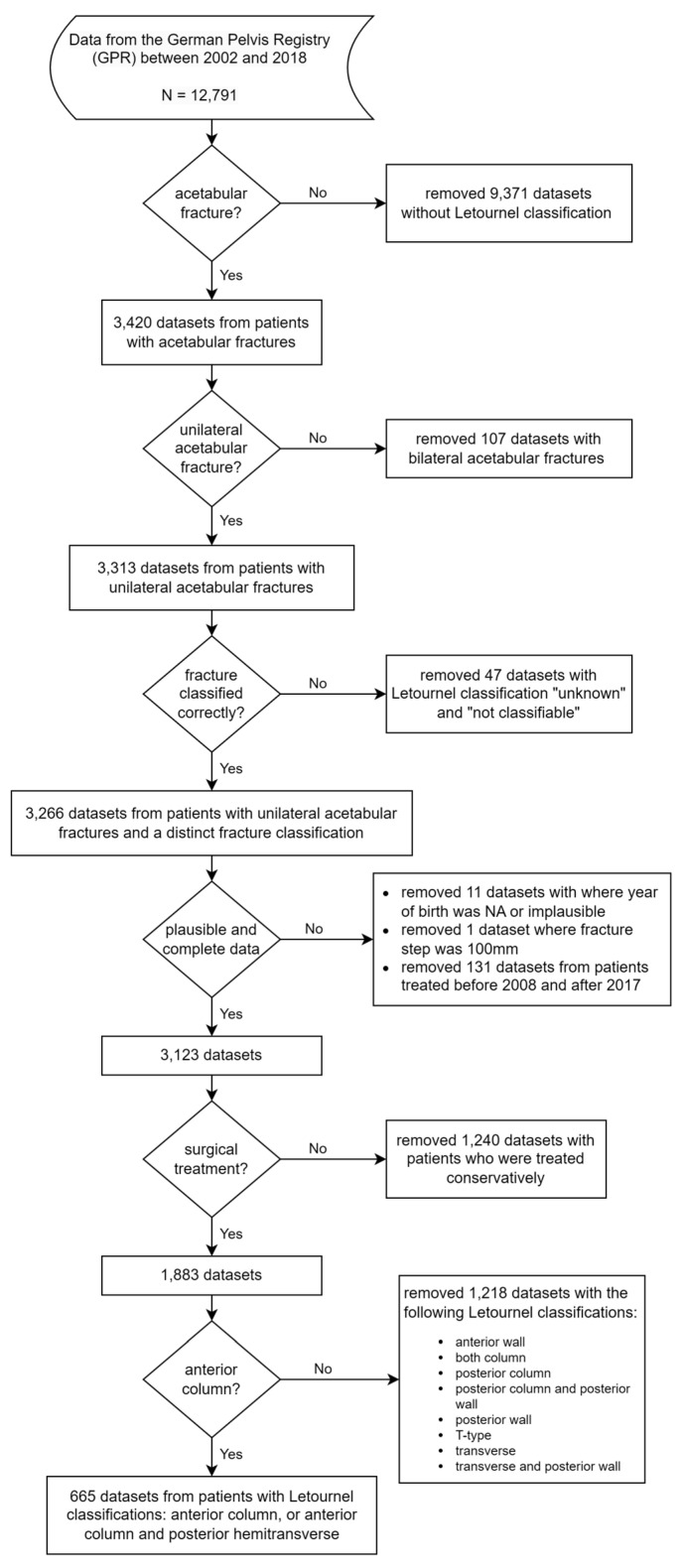
Cohort definition (GPR = German Pelvis Registry).

Between 2008 and 2017, 3,123 cases of acetabular fractures were retrospectively analyzed. The exclusion criteria were conservatively treated patients and patients with fracture types according to Letournel, other than anterior column and anterior column/posterior hemitransverse. Furthermore, all fracture types that required a posterior Kocher–Langenbeck approach were excluded because the complications of using this approach differ substantially from those of anterior intrapelvic approaches [25,26]. The German Pelvis Registry (GPR) dataset provided to our study group by the German Pelvic Multicenter Study Group included data from 2002 to 2018. We excluded 129 datasets between 2002 and 2007 and two datasets collected in 2018 because at that time, the data quality was insufficient, and many datasets were incomplete. In 2018, there was a change in the General Data Protection Regulation that resulted in almost no data recording. A total of 665 patients were included in the final analysis: 317 patients under the age of 65 and 348 patients over the age of 65. A flowchart illustrating the process of cohort definition is depicted in Figure 1.

The type of fracture was analyzed using imaging computed tomography (CT) and categorized based on the Letournel classification [27]. Fractures were then divided into those involving the anterior aspects (anterior wall, anterior column, anterior column posterior hemitransverse, transverse, two column, t-type) vs. the posterior aspects of the acetabulum (posterior wall, posterior column).

Because acetabular fractures of the posterior wall and posterior column tend to result in subluxations or posterior dislocation of the hip joint, an immediate reduction, and if instability remains, osteosynthesis, is required. In that case, surgery is mandatory and no delay in timing should be accepted. Therefore, all those patients were excluded from this study.

### 2.2. Determining and Comparing the Fracture Type, Reduction Quality, Demographic Data, Complication Rate, and Time from Injury to Surgery

In addition to the demographic data and fracture type, the following data were analyzed: time from injury to operation, complications, reduction quality, and length of hospital stay. Furthermore, the cohort was divided into 3 treatment groups: surgery within 2 days of the accident, surgery within 2–4 days of the accident, and surgery more than 4 days after the accident. In addition, because the geriatric population is of special interest regarding the timing of surgery, we divided the cohort into two groups of patients below and above 65 years of age.

For better comparability, we focused on the geriatric patient group with isolated acetabular fractures involving the anterior aspects of the acetabulum.

To evaluate the reduction quality after operative treatment, we used the Matta grading system, which is based on the maximal residual displacement of the fracture in mm and categorized as anatomical (0–1 mm), imperfect (2–3 mm), or poor (>3 mm) [28].

### 2.3. Statistical Analysis

All statistical analyses and figures were compiled in R (R Core Team, version 4.2.3 running on Windows 10 x64). Descriptive statistics for continuous variables and frequency counts for categorical variables were calculated. To assess the differences between the groups based on the injury pattern, we used one-way analysis of variance (ANOVA) for age and Pearson’s chi-square test for the variables sex and surgical access (ventral vs. dorsal). The differences between age groups (below 65 years vs. 65 and above) and groups based on the timing of surgery (within 2 days, between 2 and 4 days, more than 4 days post-injury) were assessed using Pearson’s chi-square test or independent t-test, as appropriate. The level of significance was set at α < 0.05.

## 3. Results

From 2008 to 2017, a total of 665 patients (317 patients < 65 and 348 ≥ 65 years of age) were enrolled in our study and retrospectively analyzed. Table 1 shows demographic data (sex and age) based on injury pattern (isolated, multiple injuries, complex or polytraumatized). The vast majority of the patients were male (512 vs. 153). In 68% of the patients, the acetabulum fracture occurred as a monotrauma, whereas 32% were multiply injured or polytraumatized. Complex injury patterns according to the registry database were associated with aggravating factors, such as vascular injury or neurological impairments. Demographic data are shown in Table 1.

### 3.1. Fracture Type According to Letournel

**Figure 2:**
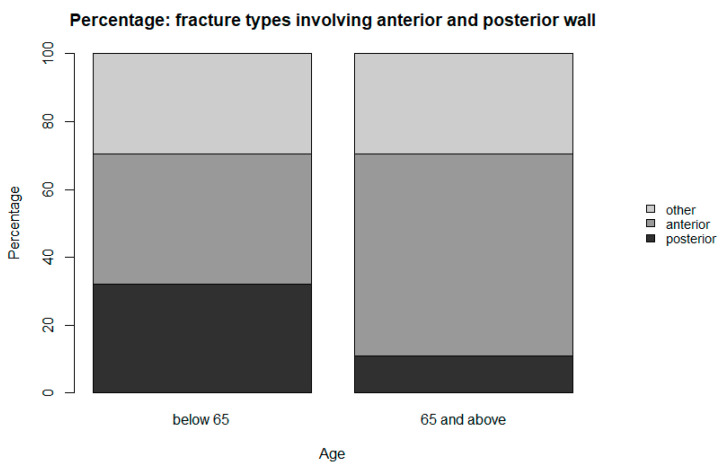
Percentage of fracture types involving anterior and posterior aspects of the acetabulum.

As mentioned above, fracture types involving the anterior column and anterior column with posterior hemitransverse are of special interest. Figure 2 and Table 2 below show the high incidence of these fracture types in the geriatric population and the distribution of the fracture types according to patient age.

### 3.2. Time until Operation

**Figure 3:**
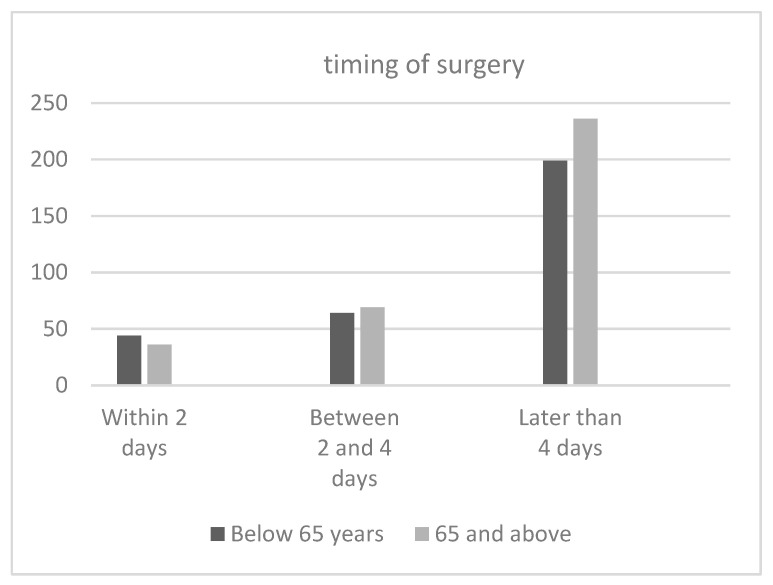
Timing of surgery in each age group.

According to the literature we found, the majority of patients in both age groups underwent an operation more than 4 days after the trauma, as shown in Figure 3. There was no significant difference in the age groups above and below 65 years of age. Only 10,3% (36/348) of geriatric patients underwent an operation within the first 48 hours (Table 3).

### 3.3. Complications

**Figure 4:**
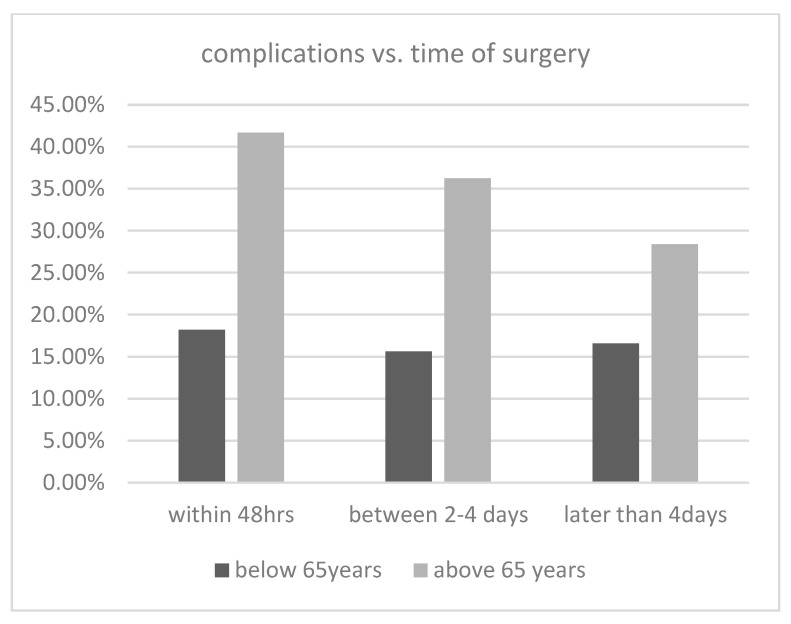
Incidence of complications for each age group and surgery date.

The detailed complications, as recorded in the registry, are shown in Table 4. Many common complications in geriatric patients, for example, urinary tract infections, pneumonia, and delirium, are summarized among “others” in the registry. The overall complication rate was approximately 16.9% in the group below 65 years of age, varying from 15.6% to 18.18% depending on the timing of surgery. In the age group above 65 years of age, the overall complication rate was approximately 30.75%, varying from 28.39% (more than 4 days) to 41.67% within 48 hours, which was a significant difference in comparison with the overall population (Figure 4 and Table 4).

### 3.4. Reduction Quality Postoperatively

**Figure 5:**
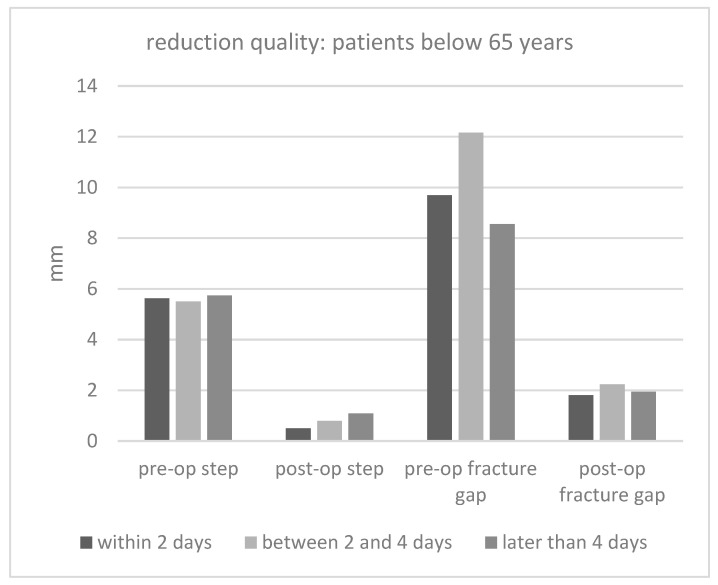
Reduction quality (fracture steps and gaps) in patients below 65 years of age for every surgery date (mm = millimeter).

**Figure 6:**
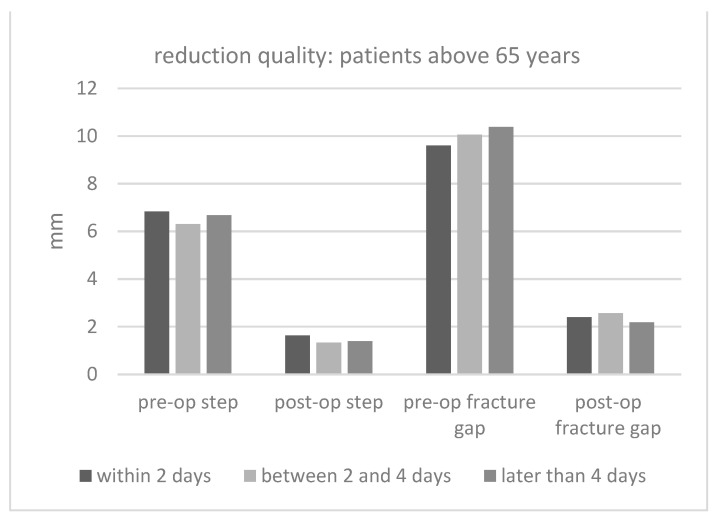
Fracture steps and gaps in patients above 65 years of age for every surgery date (mm = millimeter).

Figure 5 and Figure 6 show the pre- and post-op fracture steps and gaps. Obviously, the operation decreased the steps and gaps significantly. When analyzing the entire dataset, there was a tendency toward a worse reduction quality in patients who underwent an operation after more than 2 days. However, there was no statistical significance.

## 4. Discussion

Acetabular fractures in young people often result from high-energy trauma, mainly traffic accidents. In this age and patient group, however, an isolated acetabular fracture rarely occurs [29,30,31]. There are often aggravating concomitant injuries to the head, chest, abdomen, or lower extremities, and their management is critical to the outcome. Often, these injuries also determine the time for definitive osteosynthesis of the acetabular fracture [31]. These patients are often polytraumatized, and the damage control principle applies to surgical therapy. With the exception of hip joint dislocations, acetabular fractures are often subordinate in terms of surgical urgency and are only addressed surgically when the patient has been sufficiently stabilized [21,32]. These facts may have led to the principle of delayed operative treatment for acetabular fractures.

However, for several years, a significant increase in elderly patients suffering from acetabular fractures has been observed [9,10,33]. In this cohort, individual injuries, especially, lead to fractures involving the anterior acetabular column [9,33]. The treatment of these patients is more difficult because comorbidities are frequently present. Age-related malnutrition, osteoporosis, polypharmacy (especially blood thinners), and cognitive impairment are common. These are contributing factors for an increased rate of complications [33,34]. This group of patients also frequently suffers from fractures of the proximal femur. Numerous studies have shown that prompt surgical therapy reduces mortality after a proximal femur fracture in all age groups [15,19,20,35,36]. Accordingly, it is reasonable to conclude that this should also be the case for acetabular fractures. However, this consideration is contradicted by the subjective experience of numerous acetabulum surgeons, who have observed that the early surgical treatment of acetabular fractures is associated with an increased rate of intraoperative and perioperative complications, such as an increased risk of bleeding. Therefore, some authors recommend a delayed surgical treatment of at least 72 h after the trauma [37].

The current data regarding the correct timing for surgery are extremely limited. Thus, establishing recommendations is only possible with reservations. Seilern and Aspang et al. examined the correlation between the timing of surgery after trauma and the occurrence of postoperative complications in geriatric patients [38]. In their study, 51 patients were retrospectively analyzed. They found that geriatric patients who underwent an operation after more than 48 h had a 5-fold increased risk of developing postoperative major complications, such as myocardial infarction, pulmonary artery embolisms (LAEs), deep venous thrombosis, ARDS, or renal failure, as well as less serious complications, such as pneumonia, delirium, urinary tract infections, or superficial wound infections, as compared to the group that underwent an operation within 48 hours. In addition, early-operated patients had a 7-day shorter hospital stay [38]. A review from Mansour et al. found that operative stabilization within 48 h improves repositioning quality and correlates with a statistically nonsignificant shorter surgery time. Furthermore, a shorter duration of surgery time was found to reduce postoperative wound infection rates [20]. Another retrospective study of Devaney et al. found advantages for the early definitive surgical stabilization of pelvic ring injuries but could not correlate the effect on acetabular fractures [39]. According to Plaisier et al., patients who underwent acetabular reconstruction within 24 h of injury had reduced organ dysfunction and improved functional outcomes compared with those who received delayed treatment [15]. This was confirmed by Vallier et al., who found that in 359 patients, surgery within 24 h reduced the risk of morbidity and shortened the time spent in the intensive care unit [40]. Furthermore, Vallier et al. published a protocol to determine the ideal timing of surgery for the stabilization of fractures in resuscitated patients [40]. The authors recommend using lactate levels (<4.0 mmol/L), pH (≥7.25), or base excess (≥5.5 mmol/L) as guidelines. If these parameters show normal results, osteosynthesis could be performed within 36 h, which would reduce the risk of postoperative complications, such as pneumonia, acute renal failure, infections, sepsis, and total hospital stay. However, in a retrospective analysis of 183 patients, Glogovac et al. found that surgery within 48 h did not benefit the outcome and recommended a patient-specific decision for the timing of surgery [41]. Moldovan et al. analyzed routine blood-derived biomarkers in geriatric patients with hip fractures to evaluate the severity of the injury as a predictor for perioperative complications and mortality. These findings can also be considered when determining the optimal time for surgery [42].

Unfortunately, the current literature generally lacks consistency in defining the time period that constitutes early surgical intervention. Whereas some studies define early surgery as being performed within the first 7 days after the accident [19] others specify a timeframe of 48 hours [35,36]. Furthermore, due to the different types of fractures, comparability is limited. Unlike pelvic ring fractures or proximal femur fractures, the classification of acetabular fractures is complex. The five simple and five combined fracture types may not accurately reflect the severity of the injury or the extent of dislocation. The treatment algorithms for these injuries range from conservative to purely dorsal approaches, as well as intra- and extra-pelvic anterior approaches, or a combination of these procedures. This is why comparing the results of individual studies is challenging, and it is not possible to find prognostic factors or recommend treatment based only on retrospective single-center studies. Therefore, only multicenter studies or a registry database can provide sufficient information.

In our cohort study, we analyzed all surgically stabilized acetabular fractures from the German Pelvic Registry with the two fracture patterns of interest (anterior column and anterior column/posterior hemitransverse) from 2008 to 2017. This study revealed that the complication rate remained constant regardless of the timing of surgery, with no significant differences observed. However, upon analyzing the subgroups, notable findings emerged. We found a significant increase in the complication rate among patients over the age of 65, compared with the overall dataset. The complication rate was twice that of patients below 65 years. Moreover, there were significant differences in the types of complications observed. Bleeding complications were more prevalent in younger patients, whereas thromboembolisms were more frequent in the elderly. However, these findings were not statistically significant. Furthermore, the most important prognostic factor after acetabular fracture is the restoration of joint congruence with as few steps and gaps as possible [43,44], and acetabular revision surgery due to post-traumatic osteoarthritis might be challenging due to severe bone loss [45]. Regarding joint congruency, there was no significant disparity observed in the timing of surgery (<2 days, 2–4 days, >4 days). However, patients who underwent surgery more than 48 h after the trauma tended to have more steps and gaps, as indicated by various studies [13,44]. This is understandable, assuming high-energy trauma resulting in substantial fracture dislocation accompanied by severe injuries. Therefore, severe and highly dislocated fractures are often challenging to reduce regardless of the timing of surgery.

Our study has certain limitations that should be acknowledged. Although the registry data provide some insights, the small patient sample in each subgroup poses challenges when interpreting the results. In Germany, approximately 8,142 cases of acetabular fractures are reported annually by the Federal Statistical Office (Statistisches Bundesamt Deutschland) [11].

The registry data contain approximately 3,500 cases of acetabular fractures, out of which 1,883 underwent surgical treatment. However, these data span over a period of 13 years, from 2004 to 2017. Hence, it is evident that the registry data are incomplete, although there has been a noticeable improvement in the recording of acetabular fractures in recent years.

The subgroup analysis further reduces the sample size. Only 36 patients, representing approximately 11.5% of surgically treated patients over 65 years of age, received surgical treatment within the first 48 h. This corresponds to only 5,4% of the entire patient cohort (ant. column and post. hemitransverse). The majority of 67% (i.e., 236/348 patients) of geriatric patients underwent surgery more than 4 days after the injury, representing approximately 35% (236/665 patients) of the entire cohort.

Therefore, the statistical data on complication rates should be viewed critically because of the small sample size as individual outliers can have a significant impact on the results.

In the dataset, the majority of complications are summarized as other, which makes a detailed analysis of these findings impossible. Secondly, the retrospective design and the reliance on existing data without knowledge of confounding variables is challenging. Moreover, a statement about functional outcomes is missing. Additionally, there is no follow-up period, which makes interpreting the impact of the timing of surgery on long-term results unfeasible.

Important information, such as the recording of anticoagulant therapy, quantification of blood loss, number of blood products given, and severity of thromboembolism, is not provided.

Because there are no data on the amount of blood loss or the number of blood products given, this study lacks significant facts when interpreting the registry data regarding surgical timing.

## 5. Conclusions

Based on the data available, the perfect time for surgery of acetabular fractures remains unclear. It can cautiously be stated that early surgery within 48 h following acetabular fractures does not increase the complication rate in both—the overall group and the geriatric subgroup, at least for the above-mentioned complications (blood loss, infection, implant failure). However, in the geriatric population, we believe that there are more common complications, such as perioperative delirium, that are relevant to the patient’s outcome. In further studies, these complications need to be evaluated. Also, the exact intraoperative blood loss and the impact of blood thinners are of immense interest.

As far as reduction quality is concerned, early stabilization seems to be beneficial, although the comparability of the fractures is difficult to register.

Functional scores to determine the morbidity of these patients with respect to the procedure performed or the approach chosen should be included in further studies. Contributing cofactors (e.g., blood-derived biomarkers, serum lactate levels, etc.) can be evaluated to further refine the optimal time for surgery.

In general, the timing of surgery alone cannot be considered as the only critical factor in determining the success of the procedure. Thorough preoperative planning and choice of the best team are more important than focusing solely on scheduling surgery as soon as possible. However, it is crucial to recognize emergency indications and initiate primary treatment on the day of the accident in certain cases.

## Figures and Tables

**Table 1:** Patient characteristics based on injury pattern (isolated, multiple injuries, complex or polytraumatized).

Injury Pattern	Age: Mean (SD)	Sex	
		Female	Male
isolated	67.46 (16.13)	111	342
multiply injured	59.32 (15.63)	20	106
polytrauma	46.85 (19.05)	17	43
complex	54.31 (19.10	5	21
*p*	<0.001 a	0.126 b	

Abbreviations: SD = standard deviation, a = one-way ANOVA (one-way analysis of variance); b = Fisher’s exact test.

**Table 2:** Fracture type versus patient age.

Fracture Type	below 65 Years	above 65 Years
ant. column	141	133
ant. column and post. hemitransverse	176	215
total	317	348

Abbreviations: (ant. = anterior; post. = posterior).

**Table 3:** Age group versus timing of surgical intervention.

Operation	within 2 Days		between 2 and 4 Days		more than 4 Days	
below 65 years	44	13.8%	64	20.2%	199	62.8%
above 65 years	36	10.3%	69	19.8%	236	67.8%
*p*	*p* = 0.308					

**Table 4:** Complications at the time of surgery based on timing of surgery and age group.

Complications	within 2 Days		between 2 and 4 Days		more than 4 Days	
	below 65 Years	above 65 Years	below 65 Years	above 65 Years	below 65 Years	above 65 Years
thrombosis	0	2	1	1	6	6
embolism	0	0	1	1	1	4
ARDS	0	0	0	0	1	2
MOF	0	1	1	0	0	0
nerve palsy	5	1	1	4	4	5
infection sup.	0	2	0	0	2	3
infection deep	0	2	1	4	4	7
bleeding	0	0	0	5	1	6
hematoma	0	1	1	2	1	5
seroma	0	0	0	0	0	0
wound healing disorder	0	1	0	0	2	0
implant loosening	1	1	1	1	1	0
implant failure	0	0	0	0	0	2
secondary dislocation	0	0	0	1	0	1
other	2	4	3	6	11	26

Abbreviations: (sup. = superficial; MOF = multiorgan failure; ARDS = acute respiratory distress syndrome).

## Data Availability

Data are unavailable due to privacy or ethical restrictions.
